# Settings and artefacts relevant for Doppler ultrasound in large vessel vasculitis

**DOI:** 10.1186/s13075-017-1374-1

**Published:** 2017-07-20

**Authors:** L. Terslev, A. P. Diamantopoulos, U. Møller Døhn, W. A. Schmidt, S. Torp-Pedersen

**Affiliations:** 1 Center for Rheumatology and Spinal Diseases, Copenhagen University Hospital, Rigshospitalet, Copenhagen, Denmark; 2 Department of Rheumatology, Hospital of Southern Norway Trust, Kristiansand, Norway; 3 Medical Centre for Rheumatology, Immanuel Krankenhaus, Berlin, Germany; 4 Department Radiology, Copenhagen University Hospital. Rigshospitalet, Copenhagen, Denmark

**Keywords:** Ultrasound, Doppler ultrasound, Large vessel vasculitis, Giant cell arteritis

## Abstract

Ultrasound is used increasingly for diagnosing large vessel vasculitis (LVV). The application of Doppler in LVV is very different from in arthritic conditions. This paper aims to explain the most important Doppler parameters, including spectral Doppler, and how the settings differ from those used in arthritic conditions and provide recommendations for optimal adjustments. This is addressed through relevant Doppler physics, focusing, for example, on the Doppler shift equation and how angle correction ensures correctly displayed blood velocity. Recommendations for optimal settings are given, focusing especially on pulse repetition frequency (PRF), gain and Doppler frequency and how they impact on detection of flow. Doppler artefacts are inherent and may be affected by the adjustment of settings. The most important artefacts to be aware of, and to be able to eliminate or minimize, are random noise and blooming, aliasing and motion artefacts. Random noise and blooming artefacts can be eliminated by lowering the Doppler gain. Aliasing and motion artefacts occur when the PRF is set too low, and correct adjustment of the PRF is crucial. Some artefacts, like mirror and reverberation artefacts, cannot be eliminated and should therefore be recognised when they occur. The commonly encountered artefacts, their importance for image interpretation and how to adjust Doppler setting in order to eliminate or minimize them are explained thoroughly with imaging examples in this review.

## Background

Diagnosis and monitoring of large vessel vasculitis (LVV) are part of most rheumatology clinics. LVV includes giant cell arteritis with and without large vessel involvement and Takayasu arteritis. Giant cell arteritis is seen in people aged >50 years, with a predilection for the temporal and other extracranial arteries such as the axillary and subclavian arteries. Takayasu arteritis appears in younger people with disease onset before the age of 40 years. A distinctive part of LVV is an increase of the intima-media vessel wall thickness, which may result in stenosis or even occlusion (altering the flow profile and changing the blood velocity in the affected areas of the vessels).

Several publications have highlighted the use of ultrasound for diagnosing LVV and monitoring disease activity [1–6] and in some institutions it has even substituted for temporal artery biopsy [6, 7]. Some of the vasculitis features, such as increased intima-media vessel wall thickness, are diagnosed with greyscale (GS) ultrasound alone but Doppler ultrasound plays a role by aiding in visualising the vessel and the wall swelling and pinpointing the areas of stenosis or occlusion and, hence, the importance of changes in flow velocity and direction.

Though Doppler ultrasound is used routinely in most rheumatology departments for patients with arthritis, the Doppler settings for ultrasound in large vessels and the resulting artefacts are very different. In arthritic joints the type of vascularization is characterised by slow flow, and the vessels of interest are invisible without the Doppler mode with minimal movement of the vessel wall during the cardiac cycle. The type of flow in arteries affected by LVV is fast flow. Large vessels have considerable movement of the vessel wall during the cardiac cycle. These differences in flow for arthritis and vasculitis have an impact on the optimal Doppler settings for each.

The correct interpretation of flow requires knowledge of physical and technical factors influencing the Doppler signal. Artefacts caused by physical limitations of the modality or inappropriate equipment settings may result in flow characteristics that differ considerably from the actual physiologic situation, consequently leading to misinterpretation of flow information.

The different factors and their impact on flow are described in the following sections.

## The ability to detect flow—the Doppler shift

The ability to detect flow is caused by the Doppler shift, which is a change in the wavelength (frequency) of sound resulting from motion of a source, receiver or reflector. As the ultrasound transducer is both a stationary source and a receiver of sound, the Doppler shift arises from reflectors in motion—for all practical purposes the erythrocytes [8].

When the ultrasound pulse is reflected from moving erythrocytes, two successive Doppler shifts are involved. First, the sound from the stationary transmitter—the transducer—is received by the moving erythrocytes—the receiver in motion. Second, the erythrocytes act as moving sources of ultrasound as they re-eradiate the ultrasound wave back toward the transducer—the emitter in motion. These two Doppler shifts account for the factor 2 in the Doppler equation:

$$ {f}_D={f}_t-{f}_r=\frac{2{f}_t v \cos \theta}{c} $$

where *f*_*D*_ is the Doppler shift, *f*_*t*_ is the transmitted frequency, *f*_*r*_ is the received frequency, *v* is the blood velocity, θ is the insonation angle (angle between the ultrasound beam and the blood flow) and *c* is the speed of sound. The Doppler shift is thus directly proportional to the velocity of the flow (*v*), cosine (cos) to the insonation angle (θ), and the transmitted frequency of the ultrasound (*f*_*t*_) [9]. The Doppler used is called pulsed Doppler. With a series of pulses, the phase of the returning signals is compared to the phase of the emitted signal. A change in phase translates to a change in frequency—e.g. when the returning signal is compared to the emitted, returning wave tops will not correspond to the emitted wave tops because the distance between the tops has changed. The number of these pulses per second is called the pulse repetition frequency (PRF).

### The insonation angle and blood velocity

**Fig. 1:**
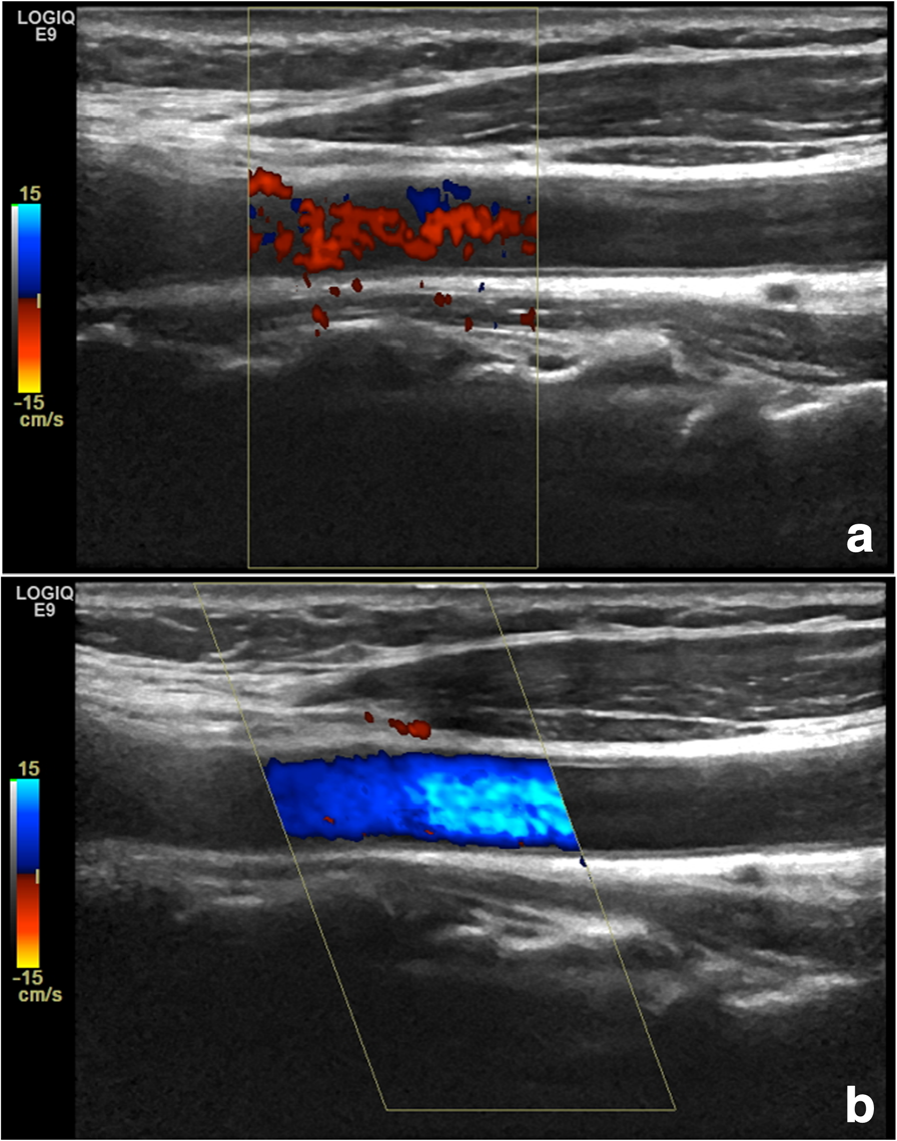
The insonation angle is the angle between the path of the Doppler pulses and the direction of flow in the vessel as indicated by the orientation of the Doppler box. When this angle is 90° (*top*), there will be no frequency shift because cos(90°) = 0. With angle correction in this carotid artery more of the flow becomes detectable (*bottom*)

The insonation angle is the angle between the path of the Doppler pulses and the direction of flow in the vessel. When this angle is 90°, there will be no frequency shift because cos(90°) = 0 (Fig. 1). The maximum frequency shift of a given vessel is obtained when the direction of flow matches the direction of the Doppler pulses (directly towards or away from the transducer) giving an insonation angle of 0° or 180°, resulting in cos(θ) = ±1.

When measuring the velocity of the blood flow spectral Doppler is used, and the Doppler equation is rearranged:

$$ {f}_D=\frac{2{f}_t v \cos \theta}{c}\iff v=\frac{f_D c}{2{f}_t \cos \theta} $$

As seen, blood velocity can only be determined if the insonation angle is known and entered into the machine—the angle correction. In synovial flow where the insonation angle is unknown as the vessels cannot be seen, the velocity is calculated based on the assumption that the Doppler angle is zero. Therefore, the displayed velocities are most often incorrect. However, the large(r) vessels assessed in LVV are easily seen and angle correction is possible, allowing for correct velocity calculation. Angle correction can only be made in spectral Doppler.

In arthritis, the presence of flow is important, not direction nor velocity, and angle correction has no relevance. In LVV, correct blood velocity is important for diagnosing the degree of stenosis [10, 11]**.**

## Doppler modalities and choice of Doppler mode

There are three Doppler modes: colour Doppler (CD), power Doppler (PD), and spectral Doppler. The two “colour” Doppler modalities—PD and CD—show different aspects of the flow detected superimposed on the GS image.

The Doppler analysis is carried out in the Doppler box, defining the region of interest. Inside the box, the image is divided into small cells, each behaving like an independent Doppler gate with its own Doppler analysis. For both colour modalities, a Doppler shift (change in frequency) has to be detected before any Doppler information is displayed. The difference between the two modalities is the way the Doppler information is displayed and not the way the Doppler shift is detected. The ability to detect the Doppler shift determines the machine’s Doppler sensitivity, which may be different for CD and PD and has to be determined in practice [12].

### Colour Doppler

In CD, the mean frequency shift for each cell inside the Doppler box is displayed as a colour according to a colour code. The colours that arise from the detected Doppler shifts indicate the qualitative direction of flow and also relative velocities and is an image of the mean blood velocity. The colour “red” is most often by default set to flow towards the top of the image and blue towards the bottom. Different hues of red and blue indicate different velocities (in reality different frequency shifts).

CD displays the direction and velocity of the flow and is the recommended Doppler modality for LVV.

### Power Doppler

PD displays the energy in each cell from all the moving erythrocytes—neither direction nor velocity. Disregarding direction of flow (negative or positive frequency shift) and velocity (high- or low-frequency shift) the power (energy) of the many different frequency shifts inside a cell are added to form the PD signal. The brighter the colour the higher the energy. In PD, the power of the signal from each point relates to the number of moving erythrocytes in that sample volume, which means it depicts the amount of blood moving in each cell. PD images may be regarded as images of the detected blood pool.

PD does not detect direction nor velocity and is not recommended for LVV.

### Spectral Doppler

In spectral Doppler, a Doppler line is displayed in the GS image indicating the path of the Doppler beam. This line may be vertical or angled relative to the GS image. This is called Duplex ultrasound (GS image and spectral Doppler) and if PD/CD is present as well, it is called triplex ultrasound.

**Fig. 2:**
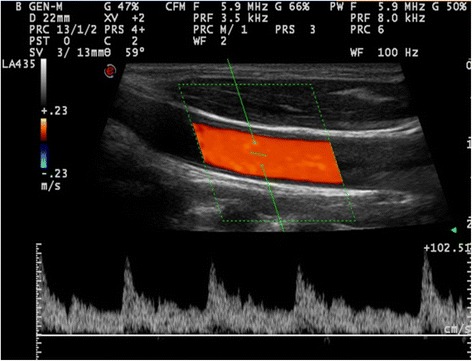
In spectral Doppler, the flow is displayed as a spectral waveform of changes in velocity during the cardiac cycle. The flow velocity is on the vertical axis with the time on the horizontal axis. The image is from a carotid artery

On the Doppler line the measurement area—the gate—is bordered by two parallel lines. The gate (area between the two lines) can be moved up and down on the line and adjusted in size. With spectral Doppler, the detected Doppler shifts within the gate are plotted against time, and the blood velocity throughout the cardiac cycle is shown on a graph. To display the correct blood velocity, angle correction must be made. When angle correction is activated a line appears centrally in the Doppler gate—the operator aligns this line with the orientation of the vessel and the machine then determines the insonation angle (see the rearranged Doppler equation)—allowing the detected Doppler shifts (kHz) to be translated into velocities (m/s). The flow is displayed as a spectral waveform of changes in velocity during the cardiac cycle. The flow velocity is the vertical axis with the time on the horizontal axis (Fig. 2).

Inside the vessel, the erythrocytes travel with different velocities; the highest velocities are found in the centre of the vessel and the lowest velocities nearest the wall. On the graph, the velocities throughout the cardiac cycle are shown with a line with a certain thickness. The thickness of the line displays the distance between the fastest and the slowest flow inside the Doppler gate.

With spectral Doppler it is possible to display the flow velocities in the vessels.

## Optimal Doppler settings

Several adjustable parameters may improve the Doppler findings and are discussed in the following sections.

### Doppler frequency

**Fig. 3:**
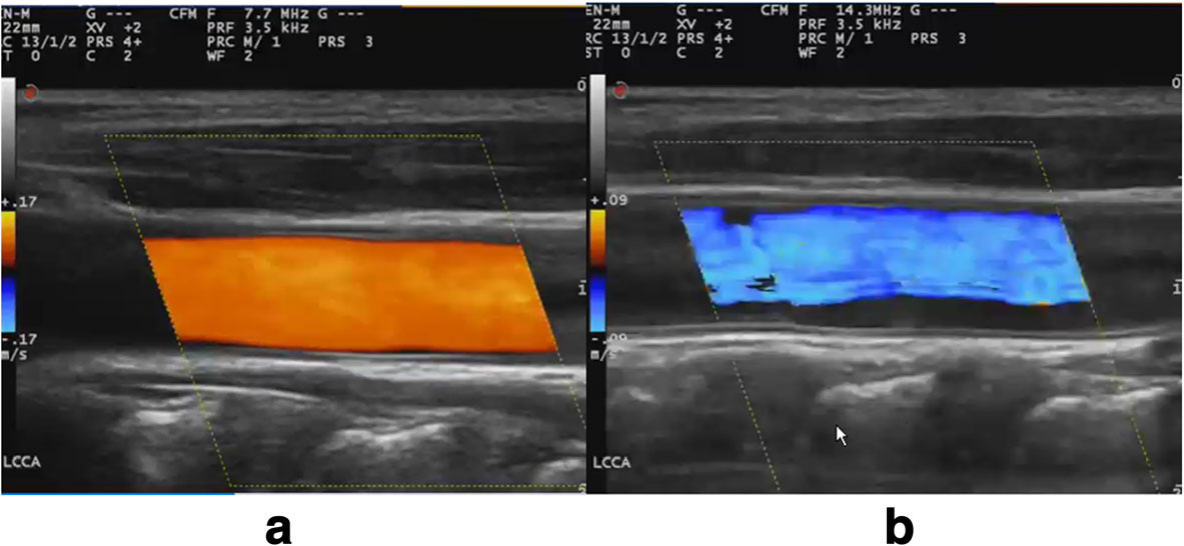
A lower Doppler frequency allows more penetration but also a lower resolution. A higher Doppler frequency gives a more detailed image of the vessels at the expense of penetration. *Left*: carotid artery with a Doppler frequency of 7.7 MHz. *Right*: carotid artery with a Doppler frequency of 14.3 MHz; this cannot penetrate well enough to give optimal flow information

The Doppler frequency at which the transducer operates is selectable. As in GS ultrasound, a lower Doppler frequency allows more penetration but also a lower resolution. Thus, higher Doppler frequency gives a more detailed image of the vessels at the expense of penetration (Fig. 3). The trade-off between penetration and sensitivity is somewhat unpredictable because resolution in this context is not an issue. An inappropriate Doppler frequency will result in an inability to detect flow.

The optimal Doppler frequency must be found in practice and not in theory.

### Colour box

The colour box is the area where evaluation of flow is performed. The numerous Doppler analyses inside the colour box are computationally demanding on the ultrasound unit. This results in a decrease of GS frame rate and the larger the colour box, the lower the frame rate. It is recommended to adjust the box so it covers the region of interest and goes to the top of the image, even though this is not as imperative as in arthritis (see Doppler artefacts relevant in fast flow).

Some vessels are horizontally orientated and almost parallel to the probe (orthogonal to the Doppler beam), which decrease the Doppler shifts and thereby the detection of flow.

To improve flow detection, it is recommended to angle the Doppler box to avoid an insonation angle close to 90° but preferably below 60^o^ [13].

### Pulse repetition frequency

PRF is the Doppler sampling frequency of the transducer and is reported in kilo Hertz (KHz). The frequency with which these pulses are emitted determines the maximum Doppler shifts obtainable. The maximum Doppler shift frequency that can be sampled without aliasing is PRF/2, called the Nyquist limit [14]. The Nyquist limit may be presented on-screen as a blood velocity (the maximum measurable velocity of blood moving directly towards or away from the transducer) or in kHz (maximum measurable Doppler shift). If the blood velocity (and thereby the Doppler shift) is above the Nyquist limit, the machine will misinterpret the velocity and direction, causing aliasing (see section on Doppler artefacts relevant in fast flow).

**Fig. 4:**
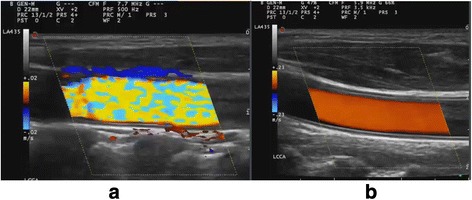
Adjustment of pulse repetition frequency (PRF) results in simultaneous adjustment of wall filters. *Left*: With a low PRF the wall filters do not remove noise from the moving vessel wall as in this carotid artery (PRF 0.5 KHz). *Right*: When a higher PRF is selected the wall filters will eliminate the noise from the moving vessel wall and only flow inside the vessel and the correct direction of flow will be shown (PRF 3.5 KHz)

When a high PRF is chosen, it is assumed that high-velocity flow is of interest. Therefore, higher wall filters that remove noise are applied (see Doppler artefacts relevant in fast flow). Adjusting PRF results in simultaneous adjustment of these wall filters (linked control). With a high PRF, the system is insensitive to lower velocities, which has the same frequency range as noise, because the higher wall filters eliminate the information. However, pulsation in large vessels makes the vessel wall move back and forth, generating noise (Fig. 4a) which disturbs the interpretation of the image if the PRF is set too low. When a higher PRF is selected the wall filters will eliminate the noise from the moving vessel wall and only flow inside the vessel and the correct flow direction will be shown (Fig. 4b).

The optimal PRF setting in LVV is the lowest possible that does not result in aliasing in a normal part of the vessel. With this setting, a stenosis will result in aliasing, which thereby becomes evident. With the PRF set too high a stenotic area may not be identified by the presence of aliasing and wall filters may eliminate true flow from the vessel lumen.

The optimal PRF will vary depending on flow velocity in the examined vessel (higher PRF in the carotid artery than in the occipital artery). The ideal PRF may also vary between patients but will often be within the same range (Table 1).

**Table 1:** How to adjust the Doppler settings in large vessel vasculitis

Flow type	The flow is fast and in vasculitis direction and velocity are important for diagnosis
PRF and wall filters	Different PRF for different vessels:
	Temporal and facial arteries, PRF range = 2–3.5 kHz
	Carotis, subclavian and axillary arteries, PRF range = 3–4 kHz
	Vertebral and occipital arteries, PRF range = 0.7–1.5 kHz
	High PRF ensures display of correct flow direction and velocity
	Wall filters are linked controls and with a high PRF, the wall filter will also be high to eliminate motion artefacts from moving arterial walls
Choice of Doppler	CD as it displays the mean blood velocity
	Spectral Doppler for indicating correct insonation angle and for calculating the correct velocity of the flow
Doppler frequency	Optimal frequency varies according to the location of the vessel
	Higher for temporal arteries lower for the larger arteries because of penetration
Gain	Gain should be adjusted according to noise level for both CD and spectral Doppler but with a minimum of blooming
Colour priority	If adjustable, then it should be kept high to ensure Doppler filling in the vessel
Focus	At the level of region of interest to ensure optimal Doppler sensitivity

**Fig. 5:**
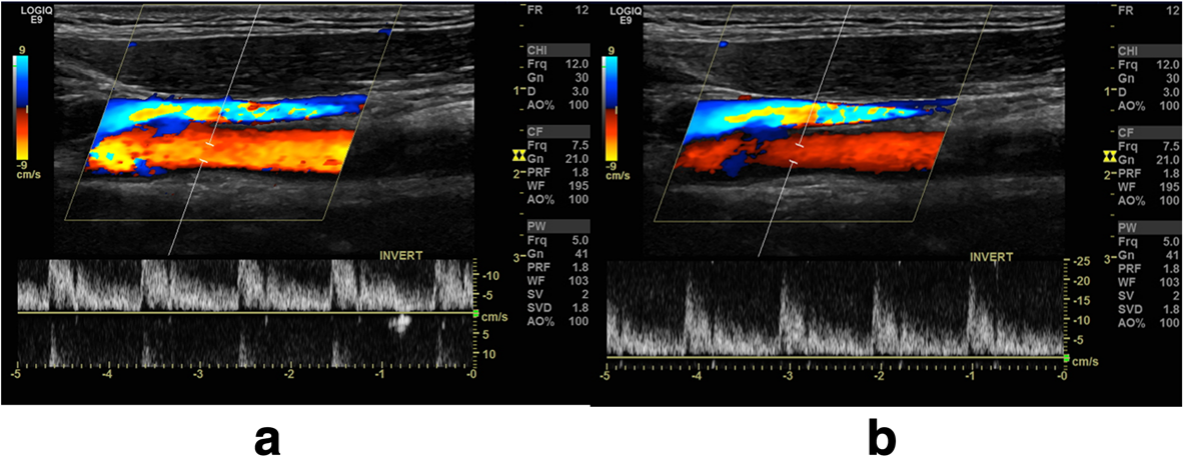
Baseline correction in spectral Doppler. The optimal PRF is without aliasing. The lower the PRF is, the more detailed (larger) the flow curve is. *Left*: When aliasing occurs it is seen on the flow curve as the top of the graph being cut off and displayed as coming from the bottom of the image. *Right*: Baseline correction in this carotid artery allows the operator to lower or elevate the baseline until there is room for the whole graph on either side of the baseline. When baseline correction is not sufficient, then PRF must be increased

The PRF in spectral Doppler is the same as in CD, where the lowest possible PRF without aliasing is preferable. The lower the PRF is, the more detailed (larger) the flow curve is. When aliasing occurs, it is seen on the flow curve as the top of the graph being cut off and displayed as coming from the bottom of the image (or vice versa). There are two ways to correct this: baseline correction or increasing the PRF—the latter will result in a smaller graph with loss of details. Baseline correction is an independent function on the machine that allows the operator to lower or elevate the baseline until there is room for the whole graph on either side of the baseline (Fig. 5).

In spectral Doppler, baseline correction eliminates aliasing and, when insufficient, PRF must be increased.

### Filters

Every Doppler instrument has high-pass filters—also called wall filters—which separate by frequency alone [15, 16]. They eliminate the lowest Doppler shifts that originate from the motion of the vessel wall and surrounding solid tissues. These unwanted shifts are called clutter or motion artefacts (low frequency and high amplitude). The machine cannot distinguish which low-frequency Doppler shifts originate from slow moving blood and which originate from tissue movement. Consequently, both will be removed when the filters are high (the PRF and wall filter are linked controls, and by increasing the PRF, the wall filters are also increased). On some machines, the filters can be adjusted manually, but the highest possible wall filter is higher for a high PRF than a low.

The wall filters should be kept high in LVV to eliminate artefacts from pulsating vessel walls.

### Doppler gain

The Doppler gain is independent of GS gain and determines the sensitivity of the system to flow. Gain is adjusted separately for CD and spectral Doppler.

**Fig. 6:**
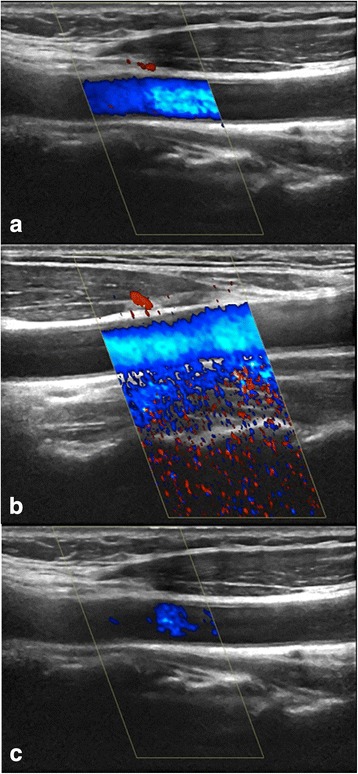
The gain determines the sensitivity of the system to flow. In this carotid artery the gain is adjusted by increasing the gain until random noise appears in the image and then lowering the gain until only a very few noise pixels are present. *Top*: Correct gain. *Middle*: Too high gain. *Bottom*: Too low gain

*CD gain* is adjusted by increasing the gain until random noise appears in the image and then lowering the gain until only a very few noise pixels are present. By lowering the gain, noise, blooming and motion artefacts are reduced but may result in only the centre of the large vessel being filled up, mimicking wall swelling, as slow flow signals alongside the vessel wall will go undetected [17, 18]. Too high gain settings result in random noise (Fig. 6) [19] or blooming where colour pixels may cover the artery walls, hiding a possible vasculitic wall swelling.

*Spectral gain* is adjusted by increasing the gain until the flow curve appears as clear as possible with potential background random noise (on the black background of the graph). Then the gain is lowered until the flow curve is clearly distinguishable from the background noise.

The gain is adjusted correctly by increasing the gain until random noise appears in the background and then lowered until only a very few noise pixels are present.

### Persistence (CD)

Persistence is a function that averages colour information over a number of frames—usually adjustable over a number of arbitrary units (e.g. 0 to 5) from minimum (no averaging) to maximum averaging. The more persistence, the more “afterglow” of the colour but at the expense of the dynamic nature of the flow.

Having a high persistence has no advantage and should be kept low to maintain the dynamic nature of the flow.

### Colour priority (threshold)

When colour information is obtained, GS information will also be present, and the machine has to decide whether to show one or the other. Colour priority (CP) is a function that determines this. It is only adjustable on some machines and on others it is a fixed setting or is handled by the machine as a linked control.

A low CP allows valid GS information to override Doppler information, helping to suppress motion and blooming artefacts in the relatively hyperechoic tissue surrounding a pulsating artery (above a certain grey level, grey overrides colour). A high CP allows Doppler information to override GS information, e.g. GS reverberation artefacts inside vessels. This function explains why some Doppler artefacts apparently prefer to appear in dark regions of the image. It also explains why GS gain may influence the amount of colour in the image, as increasing GS gain may result in more GS information being above the threshold where colour is suppressed.

**Fig. 7:**
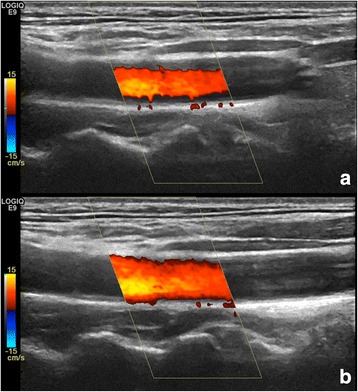
Colour priority/threshold. A low colour priority allows valid GS information to override Doppler information and a high colour priority allows Doppler information to override GS information. In the carotid artery the low colour priority results in poor filling of the vessel lumen (*top*) and should therefore be set high (*bottom*)

CP when set low will allow a relatively large vessel to be displayed without blooming or artefacts due to pulsating. However, a carotid artery may often have some degree of reverberation artefact in the lumen, and the function will then result in poor filling of the vessel lumen (Fig. 7).

CP should be adjusted to high as it may have an impact on the visualisation of flow in larger vessels.

## Doppler artefacts relevant in fast flow

### Random noise

Random noise is produced in all electrical circuits. When the gain is too high, this noise becomes visible in the CD and PD display. In the image, it is seen as colour foci appearing randomly in the image. It is easily identified as an artefact because the colour foci do not reappear in the same location as true flow does (Fig. 6b). By lowering the gain random noise will disappear.

The random noise is used to set an optimal Doppler gain see paragraph on gain adjustment.

### Aliasing

**Fig. 8:**
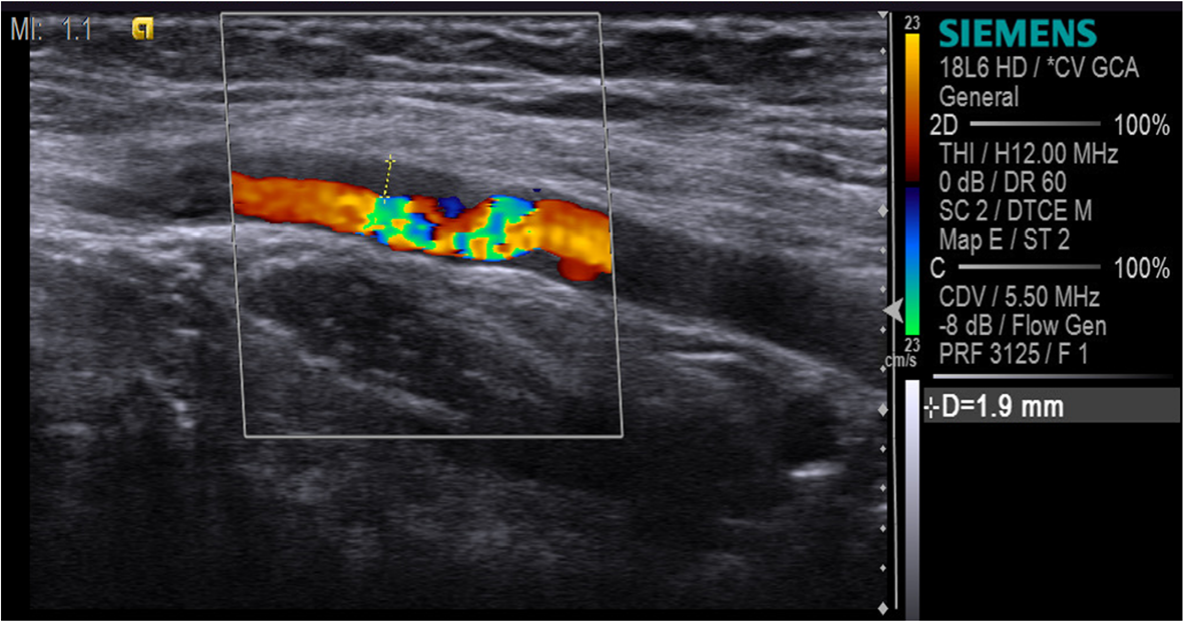
In the axillary artery the aliased signal identifies the area with stenosis because this area has the highest blood velocities. The flow is displayed with wrong direction (red instead of blue and vice versa) and velocity (the hue of the colour)

Aliasing arises when the Doppler shift of the moving blood is higher than half of the PRF (Nyquist limit). Aliased signals are displayed with the wrong direction (red instead of blue and vice versa) and velocity (the hue of the colour) (Fig. 8). Aliasing exists only in CD and spectral Doppler, and while it has no relevance in arthritic conditions, it is important in LVV to identify areas with stenosis because these areas will have the highest blood velocities. Aliasing in spectral Doppler distorts the flow information and must be corrected by adjusting the PRF before velocities can be measured.

The PRF should be adjusted so normal flow does not create aliasing. Aliasing then aids in diagnosing stenotic areas.

### Motion

The Doppler circuitry detects motion between the transducer and the tissue. When both of these are immobile, only the moving blood will generate colours in the image. Movement of the patient, transducer, or movement of the tissue or vessel wall caused by arterial pulsation during Doppler imaging give motion relative to the transducer and produce a Doppler shift [19] (Fig. 4, left). These movements are slow and produce low frequency Doppler shifts [16] that appear as random short flashes of large confluent areas of colours. Motion artefacts are easily separated from true flow signals because of their relatively large size and because they do not respect the vessel walls.

One way to avoid these low-frequency flash artefacts is to use wall filters which remove the noise together with slow flow [16]. Another option is to lower the CP; however, although this can decrease motion artefacts, it may result in poorer filling of the vessel lumen and is not recommended.

Motion artefacts should be avoided by increasing the PRF and thereby the wall filters to eliminate noise generated by the tissue movement.

### Blooming

**Fig. 9:**
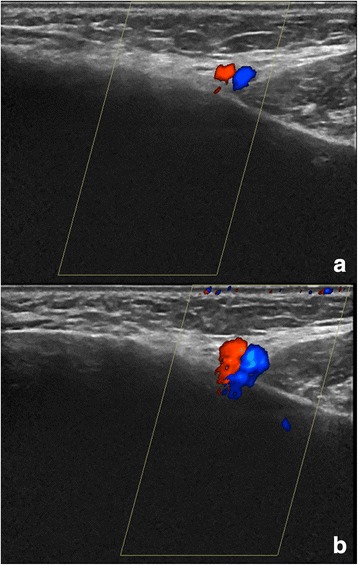
Blooming in a facial artery and vein. Blooming is gain-dependent and lowering the Doppler gain will minimize blooming artefacts (*top*), while increasing gain also increases blooming (*bottom*)

Blooming artefact describes the phenomenon that the colour “bleeds” beyond the vessel wall, making it look larger than it really is (Fig. 9). Blooming is gain-dependent and lowering the Doppler gain will minimise blooming artefacts and vice versa.

In arthritis, blooming is accepted as a systematic error as it is flow generated and, if avoided, weak flow signals will go undetected. In LVV, blooming is more problematic and must be minimised as it potentially results in colour covering a vessel wall swelling [18].

Blooming must be minimised by lowering the gain as it may disguise a wall swelling.

### Focusing

The Doppler image always uses a single focus point. On some machines the Doppler uses the same focus point as the GS image, and on others these two modalities have separate focus points. On some machines, there may be multiple focus points in the GS images while other machines only allow a single GS focus point when in Doppler mode. In the focal zone, the pulse is most narrow and has the highest spatial peak energy. Consequently, the echoes generated in the focal zone have higher amplitudes, and therefore the Doppler sensitivity is dependent upon focus positioning. Typically, focus points can be moved by the examiner in predetermined steps inside the Doppler box.

The Doppler focus point must be adjusted within the colour box to the depth of the assessed vessel.

### Mirror

**Fig. 10:**
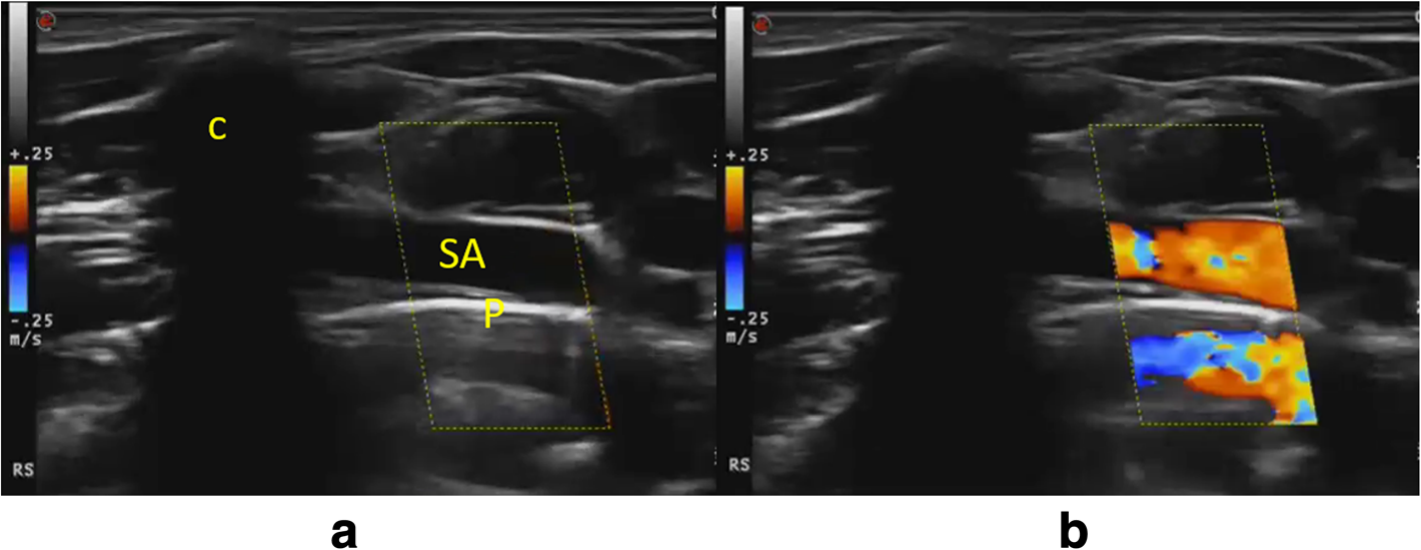
Mirror artefact. The pleura is a smooth surface with air behind that may act as an acoustic mirror. *Left*: The subclavian artery (*SA*) is seen with the pleura as a *white line* (*p*) below. *Right*: The SA is mirrored below the pleura. The clavicle (*c*) is seen casting an acoustic shadow

Any highly reflecting smooth surface may act as an acoustic mirror and the Doppler image is just as prone to mirroring as the GS image. Mirror artefacts are rare in large vessels and then the mirror surface is often air (Fig. 10). The mirror artefact is easily recognised when the true image, the mirror and mirror image all are present in the image but may be more tricky to identify when only the mirror and mirror image are present. If the artefact is investigated with spectral Doppler, it will show true flow because it is a mirror image of true flow.

The mirror artefact cannot be avoided by adjustments. With spectral Doppler, the whole Doppler spectrum may be mirrored in the posterior vessel wall, resulting in an arterial Doppler spectrum on both sides of the baseline.

### Reverberation

**Fig. 11:**
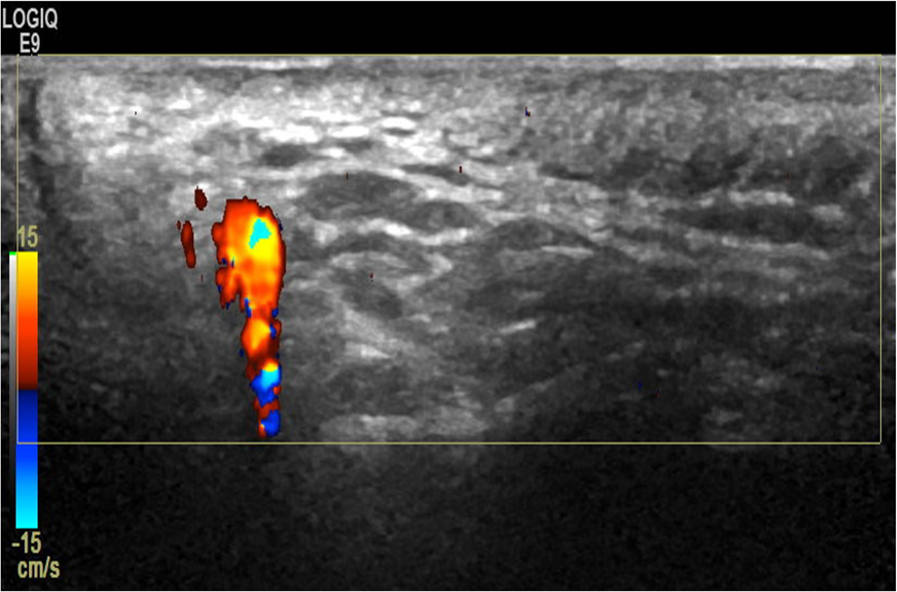
Complex reverberation artefact in temporal artery. The superficial vessel displays reverberation as a showering of colour below the vessel

The Doppler pulse behaves just as the GS pulse with respect to reverberation. A superficial vessel may be repeated lower in the image (simple reverberation) or display a showering of colour below the vessel (complex reverberation) (Fig. 11). This is especially relevant when scanning the vertebral artery where the more superficially located carotid artery may cause this artefact.

This artefact cannot be eliminated. By letting the colour box go to the top of the image, possible sources of reverberation can be detected.

### Pressure

Falsely found absence of flow may occur if the examiner presses too hard with the transducer thereby blocking the flow. This is most relevant for the temporal arteries.

It is recommended to use a sufficient amount of gel and light probe pressure.

## Discussion

In LVV, CD and spectral Doppler are promising tools to detect and monitor vessel wall inflammation and stenosis. The first step to obtaining more uniform examinations and correct interpretation of Doppler findings in LVV patients is to know how to optimize Doppler settings and become aware of frequently appearing artefacts as described in this review.

It is generally recommended to find the correct settings in healthy controls before applying the settings to a LVV patient for pathology. This is in contrast to obtaining optimal settings in arthritis, where it is recommended to adjust the settings in patients with synovial inflammation. Our suggestions to adjust settings for LVV are summarised in Table 1.

Even though Doppler ultrasound plays an important role as an aid in visualising the vessel and wall swelling and to pinpoint the areas of stenosis or occlusion, the elementary lesion of LVV is an increased intima-media wall thickness that is visualised with GS ultrasound alone, which also can be verified by a positive compression sign. The artefacts described in this paper are a natural part of Doppler scanning and should be known by all ultrasonographers. Knowledge of artefacts allows true flow to be distinguished from false flow. Noise artefacts appear randomly in the image (perhaps with a preponderance for dark regions) whereas true flow remains geographically fixed. When adjusting the gain, there is a trade-off between optimal setting and the risk of blooming that might cover wall swelling and likewise for the PRF, where the correct adjustment may be too high for optimal colour filling of the vessel, hence mimicking wall swelling.

Adjusting the many parameters of the Doppler should not be done at every examination and can be stored as presets. However, some parameters (e.g. PRF, focus and frequency) must be adjusted throughout the examination due to the different size of the vessels, the depth of their location and their flow profile.

Currently, the Outcome Measures in Rheumatology (OMERACT) ultrasound group is validating the definitions of the elementary lesions in LVV, further enhancing the use of ultrasound for vasculitis in everyday clinical use.

## Conclusions

The Doppler artefacts outlined in this review are important and relevant sources of possible misinterpretations. Artefacts cannot completely be eliminated, but can also be used constructively to optimise Doppler settings. Knowledge of Doppler physics and settings and the most frequently appearing artefacts enable investigators to make more precise and uniform interpretations of the examinations.

## Abbreviations

CD: Colour Doppler
CP: Colour priority
GS: Greyscale
LVV: Large vessel vasculitis
PD: Power Doppler
PRF: Pulse repetition frequency
